# Phytochemicals and Other Characteristics of Croatian Monovarietal Extra Virgin Olive Oils from *Oblica*, *Lastovka* and *Levantinka* Varieties

**DOI:** 10.3390/molecules20034395

**Published:** 2015-03-09

**Authors:** Mladenka Šarolić, Mirko Gugić, Emilija Friganović, Carlo Ignazio Giovanni Tuberoso, Igor Jerković

**Affiliations:** 1 Department of Food Technology, Marko Marulić Polytechnic in Knin, Petra Krešimira IV 30, Knin 22300, Croatia; E-Mails: msarolic@veleknin.hr (M.S.); mgugic@veleknin.hr (M.G.); emilija.friganovic@veleknin.hr (E.F.); 2 Department of Life and Environmental Sciences, University of Cagliari, via Ospedale 72, Cagliari 09124, Italy; E-Mail: tuberoso@unica.it; 3 Department of Organic Chemistry, Faculty of Chemistry and Technology, University of Split, N. Tesle 10/V, Split 21000, Croatia

**Keywords:** *Oblica*, *Lastovka*, *Levantinka*, virgin olive oil, headspace volatiles, phenols, GC-FID/MS, HPLC-DAD/FL

## Abstract

Virgin olive oils from the fruits of Croatian autochthonous varieties *Oblica*, *Lastovka* and *Levantinka* were characterized for the first time. Headspace volatiles were analyzed by HS-SPME/GC-FID/MS. The main volatiles were C6 compounds. The most abundant was (*E*)-hex-2-enal (62.60%–69.20%). (*Z*)-Hex-3-enal was not found in *Lastovk*a oil, while *Levantinka* oil did not contain hexanal. Tocopherols, chlorophylls and carotenoids were determined by HPLC-FL. *Levantinka* oil was characterized by the highest α-tocopherol level (222.00 mg/kg). Total phenolic contents (TPs), as well as antioxidant activity (DPPH assay) of the oils hydrophilic fractions (HFs) were assessed by spectroscopic methods. The antioxidant activity of *Oblica* oil HF was the most pronounced (0.91 mmol TEAC/kg) and the HF contained the highest TPs amount (212.21 mg/kg). HFs phenolic composition was determined by HPLC-DAD. The main identified phenols were secoiridoids dominated in *Oblica* oil: decarboxymethyl ligstroside aglycone (*p*-HPEA-EDA up to 158.5 mg/kg), oleuropein aglycone (3,4-HPEA-EA up to 96.4 mg/kg) and decarboxymethyl oleuropein aglycon (3,4-DHPEA-EDA up to 93.5 mg/kg).

## 1. Introduction

Virgin olive oil (VOO) has been a valuable vegetable oil extracted from fresh and healthy olive fruits (*Olea europeae* L.) by mechanical and other physical methods (washing, decantation, centrifugation or filtration). Nowadays, it is well-known that regular dietary consumption of VOO manifests in health benefits associated with Mediterranean diet [1]. The nutritional value of VOO arises from high level of oleic acid and from minor components such as phytosterols, carotenoids, tocopherols and hydrophilic phenols. The major phenolic compounds are oleuropein derivatives, based on hydroxytyrosol which are strong antioxidants and radical scavengers [2]. The content of phenolic compounds is an important factor to be considered when evaluating the quality of VOO, since these compounds exhibit potent antioxidant activity and contribute significantly to the extraordinary oxidation stability of VOO [3]. Chain-breaking antioxidants, such as phenolic compounds, react with lipid radicals to form nonreactive radicals, interrupting the propagation chain. In fact, these compounds are able to donate an electron or a hydrogen atom to the lipid radical formed during the propagation phase of lipid oxidation [2]. Phenolic compounds are intimately associated with the positive attributes of bitterness and pungency which are typical sensory notes of VOO obtained from the olives that are green and turning color [4]. The polar phenolic compounds of VOO belong to different classes: phenolic acids, phenylethyl alcohols, hydroxy-isochromans, flavonoids, lignans and secoiridoids [2]. The amount of polar phenols and volatile compounds in the olive oil depend on many factors such as cultivar, agronomic practices, ripeness index, fruit pre-storage, extraction, procedure, storage conditions, *etc.* [4]. Color is one of the major attributes that affects consumer perception of VOO quality. The lipophilic nature of chloroplast pigments (chlorophyll and carotenoids) determines their affinity for the oily phase and the pigments are mainly responsible for the color of VOO ranging from yellow-green to greenish gold [5]. The presence of chlorophylls and carotenoids in olive oil depends on the fruits genetic factors (olive variety), the stage of fruits ripeness, environmental conditions, production year, extraction process and storage conditions. Carotenoids, together with polyphenols and tocopherols provide oxidative stability to the olive oils and exhibit synergistic antioxidant and anticarcinogenic action at physiological concentration [6]. In addition, extra VOO is appreciated worldwide for its taste and flavor that is colored by various volatile compounds: aldehydes, alcohols, esters, hydrocarbons, ketones, furans and others [7]. It has been stated [8] that C6 compounds, the major components of VOO headspace, mainly contribute to green odor notes. These compounds are produced by lipoxygenase-mediated oxidation of polyunsaturated fatty acids containing *cis*-*cis*-penta-1,4-diene structure during the crushing and malaxation steps of the oil production [9]. Variable amounts of hexanal, hexanol and hexyl acetate derive from degradation of linoleic acid, while (*Z*)-hex-3-enal, (*E*)-hex-2-enal, (*E*)-hex-2-enol, (*Z*)-hex-3-enol and (*Z*)-hex-3-enyl acetate result from the enzymatic degradation of linolenic acid [10].

VOOs exhibit positive effect on human health as well as specific and desirable sensory properties, which is why the demand for these oils is constantly growing with request to mark their geographical and varietal origin [4]. There are about 30 autochthonous cultivars of olives in Croatia. *Oblica*, *Lastovka* and *Levantinka* are the main autochthonous varieties in Dalmatia region (south Croatia) and *Oblica* is the most abundant [11]. The aim of present research is to perform detail chemical characterization of *Oblica*, *Lastovka* and *Levantinka* VOOs (first report including statistical analysis) by: (1) determination of basic characteristics (acidity, peroxide value, K_232_ and K_270_) according to EU regulations; (2) total phenols, chlorophylls and carotenoids evaluation by UV/VIS including targeted tocopherols analysis by HPLC-FL; (3) the headspace volatiles profiling (HS-SPME/GC-FID/MS); (4) HPLC-DAD targeted phenolics analysis of the hydrophilic oil fractions (HFs) and assessing the HFs antioxidant activity (DPPH assay).

## 2. Results and Discussion

To avoid the influence of other factors, olive trees were cultivated in the same orchard under identical agronomic (*i.e.*, fertilization or irrigation) and pedoclimatic conditions and the olive fruits were picked at the same stage of ripeness and the oils were extracted with the same processing system (Section 3.1.). Therefore, it was possible to attribute the observed results exclusively to different cultivars.

### 2.1. Basic Characteristics of the Samples

The acidity, peroxide value (PV), K_232_ and K_270_ of investigated VOOs were assessed by EU method (Table 1).

**Table 1:** Basic characteristics of *Oblica*, *Lastovka* and *Levantinka* virgin olive oils (VOOs).

No.	Cultivar	Acidity ^x^ (%)	Peroxide Value ^y^ (mEq O_2_/kg)	K_232_^z^	K_270_^k^
1.	*Oblica*	0.12 ± 0.00a	2.96 ± 0.05a	1.70 ± 0.10a	0.12 ± 0.02a
2.	*Lastovka*	0.17 ± 0.00b	5.32 ± 0.04b	2.11 ± 0.40b	0.19 ± 0.01b
3.	*Levantinka*	0.13 ± 0.00c	4.00 ± 0.06c	1.93 ± 0.80c	0.15 ± 0.02c

All values are expressed as mean of triplicate determinations ± SD; ^x^ threshold value for extra VOO is ≤ 0.8; ^y^ threshold value for extra VOO is ≤ 20; ^z^ threshold value for extra VOO is ≤ 2.5; ^k^ threshold value for extra VOO is ≤ 0.22; the mean values within each column labeled with different letters are significantly different (Tukey’s test, *p* < 0.05).

The International Olive Oil Council determined acidity below 0.8% for extra VOOs (IOC/T.15/NC n° 3/Rev.7, 2012 [12]). From Table 1 it can be seen that *Oblica* variety exhibited the lowest acidity (0.12%), while the highest value was found in *Lastovka* oil (0.17%). Statistically significant differences were obtained in free acidity and peroxide value among the oils from the studied cultivars. Although significant differences were determined by Tukey’s test, the absolute values of measured acidity of all oils are very similar. Peroxide value should amount less than 20 mEq O_2_/kg for extra VOO. Determined PVs ranged from 2.96 to 4.00 mEq O_2_/kg. The lowest PV showed *Oblica* oil (2.96 mEq O_2_/kg) and the highest *Lastovka* oil (5.32 mEq O_2_/kg). Determined PVs are significantly different in all tested oils, but in absolute values they are very close. K_232_ value demonstrates conjugated dienes and their oxidation products (absorbtion at λ = 232 nm) and K_270_ value indicates conjugated trienes and secondary oxidation products (carbonyl compounds; absorbtion at λ = 270 nm). K_232_ and K_270_ values ranged from 1.70 to 2.11 and from 0.12 to 0.19 (Table 1). *Oblica* oil showed the lowest K_232_ and K_270_ values while *Lastovka* oil showed the highest values. The investigated parameters (Table 1) were within the limits of EC Reg. 1989/2003 (2003) [13] indicating the category of extra VOOs.

### 2.2. Headspace Composition

Olive oil, compared to other vegetable oils, is distinguished by a characteristic aroma. The sensory characteristics, together with high nutritional value are the main features that have resulted in the increase of VOO consumption in recent years [14]. Aroma is an important criterion for VOOs. Consequently, the identification of the compounds contributing to the aroma is considered as a key for quality and authentication control [15]. The headspace profile of tested monovarietal oils was dominated by C_6_ volatile organic compounds, mostly aldehydes (Table 2). The C_6_ compounds responsible for green and fruity VOO perception are produced through lipoxygenase pathway during the olive fruit crushing and malaxation and incorporated into resulting oil. The most abundant was (*E*)-hex-2-enal (up to 63.8%, 69.8% and 63.6% respectively). (*E*)-Hex-2-enal was the most important positive contributor of lawn perception [9].

**Table 2:** The headspace volatiles from *Oblica*, *Lastovka* and *Levantinka* VOOs determined by HS-SPME/GC-FID/MS.

No.	Compounds	RI	*Oblica* Area (%)			*Lastovka* Area (%)			*Levantinka* Area (%)		
			Min	Max	Mean ± SD	Min	Max	Mean ± SD	Min	Max	Mean ± SD
1.	Isoprene	<900	1.1	1.5	1.31 ± 0.14a	1.9	2.3	2.16 ± 0.14b	1.6	2.1	1.78 ± 0.19c
2.	Ethyl acetate	<900	0.5	0.8	0.66 ± 0.14a	1.6	1.7	1.65 ± 0.05b	0.3	0.4	0.36±0.05c
3.	Pent-1-en-3-one *	<900	1.5	1.9	1.67 ± 0.13a	3.2	4.1	3.57 ± 0.24b	2.1	2.3	2.19 ± 0.07c
4.	Pentan-3-one	<900	2.3	3.1	2.52 ± 0.24a	3.9	6.1	5.30 ± 0.75b	1.7	2.1	1.87 ± 0.16c
5.	(*E*)-Pent-2-enal	<900	0.0	0.0	0.00 ± 0.00a	0.0	0.0	0.00 ± 0.00a	0.3	0.3	0.30 ± 0.00b
6.	(*Z*)-Hex-3-enal	<900	6.2	6.9	6.48 ± 0.27a	0.0	0.0	0.00 ± 0.00b	16.2	17.0	16.64 ± 0.28c
7.	Hexanal	<900	4.1	4.5	4.40 ± 0.14a	4.8	6.2	5.31 ± 0.47b	0.0	0.0	0.00 ± 0.00c
8.	(*E*)-Hex-2-enal	<900	63.2	63.8	63.50 ± 0.24a	69.2	69.8	69.60 ± 0.19b	62.0	63.6	62.60 ± 0.49c
9.	3-Ethyloct-1,5-diene (isomer I) *	939	5.0	8.1	7.10 ± 0.88a	3.9	5.3	4.51 ± 0.53b	3.9	4.0	3.96 ± 0.05c
10.	3-Ethyloct-1,5-diene (isomer II) *	997	4.5	6.3	5.55 ± 0.67a	2.3	2.7	2.51 ± 0.11b	3.7	4.2	3.97 ± 0.16c
11.	*trans*-β-Ocimene	1054	1.0	1.1	1.06 ± 0.05a	0.1	0.4	0.21 ± 0.09b	0.0	0.0	0.00 ± 0.00c
12.	α-Copaene	1380	0.9	1.3	1.11 ± 0.11a	0.0	0.0	0.00 ± 0.00b	1.2	1.4	1.31 ± 0.09c
Total identified			(91.0%–98.2%)			(90.9%–96.1%)			(90.3%–95.7%)		

RI = retention indices on HP-5MS column; Min = minimal percentage; Max = maximal percentage; mean = average percentage; SD = standard deviation; the mean values within each row labeled with different letters are significantly different (Tukey’s test, *p* < 0.05); * - tentatively identified.

Considering other C_6_ compounds it can be seen that (*Z*)-hex-3-enal was not found in *Lastovka* oil, while *Levantinka* oil did not contain hexanal. Different values of identified C_6_ aldehydes in the samples could be due to different acyl hydrolase activity and consequently good or poor availability of free polyunsaturated fatty acids [16]. The results are in accordance with previous research [17] confirming that the cleavage by heterolytic hydroxydeperoxide lyase was the most important process (higher abundance of C6 compounds in comparison with C5 metabolites). The representatives of C_5_ compounds (Table 2) were pent-1-en-3-one, pentan-3-one and (*E*)-pent-2-enal. The percentage of pent-1-en-3-one is positively correlated with bitter taste of VOO [18]. In *Lastovka* oil the level of this compound was the highest (3.57%), while *Oblica* oil exhibited the lowest value (1.67%). In contrast to *Oblica* and *Lastovka* oils, only *Levantinka* oil contained (*E*)-pent-2-enal which can be useful potential biodiversity marker. Several hydrocarbons (such as α-copaene, *trans*-β-ocimene and two isomers of 3-ethyloct-1,5-diene) were found (Table 2). *trans*-β-Ocimene was not identified only in *Levantinka* oil, whereas, α-copaene was identified in *Oblica* and *Levantinka* oils. 3-Ethyloct-1,5-diene isomers were detected in all the oils. Statistically significant differences were found almost among all identified compounds of *Oblica*, *Lastovka* and *Levantika* VOOs.

According to [19] the most common parameters that influence the composition of VOO volatiles are: variety, growing area, the degree of maturity of fruits, harvesting method, storage conditions, storage of olive fruits after harvest, processing of fruits into oil, others. The olive fruits of different varieties grown in the same area give VOOs of different volatiles composition, but similar results were found for VOOs of the same varieties grown in different geographic areas [19] Brkić Bubola *et al.* [20] investigated volatiles and sensory characteristics of the oils from three Istrian (Croatia) olive varieties (*Buza*, *Črna* and *Rosinjola*) and found that the volatiles generated depending on the varieties which indicated a close relation with the activity of a genetically determined enzyme.

### 2.3. Targeted Tocopherols Analysis and Total Chlorophylls and Carotenoids

Tocopherols are considered as the most important lipid soluble natural antioxidants and they increase oxidation stability of the oils during storage [14]. The amount of their main component, α-tocopherol, varies by up to 300 mg/kg [21]. The concentration of β-, γ- and δ-tocopherols range from traces up to 25 mg/kg. A synergistic relationship between the antioxidant actions of some phenolics and tocopherols was demonstrated [14]. It is well accepted that tocopherols content seem to be reduced during the fruit’s ripening, refining and hydrogenation process [22].

The levels of tocopherols, chlorophylls and carotenoids determined in the oils from all varieties are shown in Table 3. The results showed the dominance of α-tocopherol in all studied oils, followed by γ-tocopherol as expected for a typical VOO. *Levantinka* oil was characterized by the highest level of α-tocopherol (222.0 mg/kg), while significantly the lowest value was found in *Lastovka* oil (177.82 mg/kg). Considering γ-tocopherol, significantly the lowest value was measured in *Lastovka* oil, whereas *Oblica* and *Levantinka* oils contained higher values (33.19 and 31.84 mg/kg, respectively). The obtained results are in accordance with our previous paper [23] on Croatian varieties *Krvavica* and *Mašnjača* as well as by Ranalli *et al.* [24] on Italian varieties *Leccino*, *Frantoio* and *Moraiolo* and Douzane *et al.* [25] on several Algerian varieties. The results agree with Perrin [26] findings that tocopherols concentration is generally greater than 100 ppm in good quality oils with α-tocopherol representing about 95% of the total fraction.

**Table 3:** The amount of α-tocopherol, γ-tocopherol, total chlorophylls and carotenoids of *Oblica*, *Lastovka* and *Levantinka* VOOs.

VOO Sample	α-Tocopherol (mg/kg)	γ-Tocopherol (mg/kg)	Chlorophylls (mg/kg)	Carotenoids (mg/kg)
*Oblica*	213.24 ± 2.2a	33.19 ± 1.86a	4.07 ± 1.21a	1.89 ± 0.37a
*Lastovka*	177.82 ± 4.0b	24.13 ± 1.18b	4.75 ± 0.61a	2.06 ± 0.21a
*Levantinka*	222.0 ± 7.17a	31.84 ± 2.16a	3.86 ± 0.85a	2.00 ± 0.27a

All values are expressed as mean of triplicate determinations ± SD; the mean values within each column labeled with different letters are significantly different (Tukey’s test, *p* < 0.05).

The color intensity of the oil is determined by changes in the source fruits’ pigment content during ripening. Although the pigment concentration in the fruit differs greatly with the variety, it decreases with ripening when chlorophylls disappear faster than carotenoids [27]. Chlorophyll and carotenoid pigments greatly influences the color of VOOs ranging from green-yellow to golden, depending on the variety and the stage of maturity [28]. As shown in Table 3, significant differences among cultivars (*p* < 0.05) were observed in α-tocopherol and γ-tocopherol among *Lastovka* VOO in comparison with *Oblica* and *Levantika* VOOs. The results show significantly higher total pigment content (chlorophylls + carotenoids) in *Lastovka* oils, with mean value of 6.81 mg/kg. *Oblica* and *Levantinka* oils contained total pigments in the amount of 5.96 and 5.86 mg/kg respectively. Chlorophylls and carotenoids ranged, respectively from 3.86 to 4.75 mg/kg and from 1.89 to 2.06 mg/kg. These findings are in agreement with previous results [29]. Moreover, Giufrida *et al.* [30] reported that the presence of the pigment in the oil depends on several factors, such as the cultivar, soil and climatic conditions, fruit ripeness and the processing procedures. Data on the pigments composition of those Croatian olive oils could be used, along with other parameters, to guarantee the genuineness and authenticity of the products. These compounds also exhibit biological and health properties and occur in the oils at concentrations which usually correlate with those of phenols and volatiles [31].

### 2.4. Total Phenolic Amounts and Targeted Phenolic Analysis

The phenolic composition of olive oil is very complex and the average concentration of these compounds depends on several factors including maturation stage, part of the fruit, variety, season, packaging, storage, climatologic conditions and the production technology [14]. VOO is well known for its high content of phenolics along with oleic acid and tocopherols that exhibit health-promoting properties [32]. Phenolic compounds influence the sensory properties (flavor, bitterness, *etc.*) of olives and the oil, and they protect against oxidative rancidity by acting as antioxidants, which are increasingly being recognized by playing a beneficial role in the diet [33].

Significant differences in total phenols (TPs) content among several HFs of the samples were observed (Table 4). *Oblica* oil exhibited the highest TPs amount (212.21 mg/kg), while *Levantinka* oil showed the lowest TPs content (144.60 mg/kg). *Lastovka* oil was quite similar to *Oblica* oil considering TPs amount (206.09 mg/kg). It is generally accepted that the TP’s level varies in the oils obtained from different cultivars and areas. Discrimination among the olive oil samples with the same geographical origin and different cultivars was possible by comparing TPs [32]. Another authors [34] claim that phenolic composition was found to be not useful in discriminating the olive oil samples due to the fact that TPs content of the oils was affected not only by the olive cultivars, but also by the climatic and environmental conditions, agronomic practice and the technological process. TPs content ranged from 50 to 1.000 mg/kg, but the values usually amount from 100 to 300 mg/kg [35] that is similar to the values found in the investigated samples. TPs content of the samples in this study could be considered as medium-high levels in accordance with previous reports [23,32,34,36].

**Table 4:** Total phenolic amounts and DPPH assay for antioxidant activities of the hydrophilic fractions of *Oblica*, *Lastovka* and *Levantinka* VOOs.

VOO Sample	Total Phenols (mg GAE/kg)	DPPH (mmol TEAC/kg)
*Oblica*	212.21 ± 7.71a	0.91 ± 0.09a
*Lastovka*	206.09 ± 13.81a	0.78 ± 0.19a
*Levantinka*	144.60 ± 2.59b	0.55 ± 0.05b

All values are expressed as mean of triplicate determinations ± SD; the mean values within each column labeled with different letters are significantly different (Tukey’s test, *p* < 0.05).

Olive oil hydrophilic extracts contain a large number of phenolic compounds including simple phenols, lignans, and secoiridoids, which exhibit antioxidant properties [37]. Among the phenolic compounds found in extra-virgin olive oils, *o*-dihydroxyphenolics are very potent antioxidants [38]. Free radicals are generated in the human body through aerobic respiration and exist in different forms, including superoxide, hydroxyl, hydroperoxyl, peroxyl and alkoxyl radicals. Natural antioxidant enzymes in healthy individuals remove these free radicals while dietary antioxidants assist the body in neutralizing free radicals. Therefore, it is important to consume foods with high contents of antioxidants, such as virgin olive oil, to reduce the harmful effects of oxidative stress [39]. Antioxidant activity measured by DPPH assay showed the highest value in hydrophilic fraction of *Oblica* oil (0.91 mmol TEAC/kg) and statistically significant lowest value in *Levantinka* oil (0.55 mmol TEAC/kg). The difference in antioxidant activity of tested oils may depend on the total phenol content in the varieties. Most of the previous studies reported strong correlation between total phenols and antioxidant capacity [23,37,39,40].

HPLC-DAD was used for targeted analysis of the polar compounds in *Oblica*, *Lastovka* and *Levantinka* VOOs. The most abundant secoiridoids of the samples (Table 5) were dialdehydic form of elenolic acid linked to hydroxytyrosol or tyrosol (*p*-HPEA) respectively assigned as 3,4-DHPEA-EDA, *p*-HPEA-EDA and 3,4-HPEA-EA. Statistically significant differences among phenolic levels were observed for the oils. *Levantinka* and *Oblica* VOOs were characterized statistically by highest amount of *p*-HPEA-EDA. *Lastovka* VOOs contained the highest amount of 3,4-HPEA-EA. 3,4-DHPEA-EDA, the dialdehydic form of decarboxymethylelenolic acid linked to hydroxytyrosol, was statistically different in all tested oils with the highest value in *Oblica* oil (85.0 mg/kg) and lowest value in *Lastovka* oil (34.4 mg/kg). Flavones, such as apigenin and luteolin, showed in total the highest value in *Oblica* oil, while the lowest value was measured in *Levantinka* oil. Lignans, such as pinoresinol, were estimated only in *Oblica* oil. These results are similar to those reported by several authors for other monovarietal VOOs [31,40,41].

**Table 5:** Targeted phenolics from *Oblica*, *Lastovka* and *Levantinka* VOOs hydrophilic fraction determined by HPLC-DAD.

No.	Compounds	RT	LOD (mg/kg)	LOQ (mg/kg)	*Oblica* (mg/kg)			*Lastovka* (mg/kg)			*Levantinka* (mg/kg)		
					Min	Max	Mean ± SD	Min	Max	Mean ± SD	Min	Max	Mean ± SD
1.	Hydroxytyrosol ^x^	10.9	0.34	1.02	4.3	6.0	5.26 ± 0.61a	5.5	9.9	7.80 ± 1.25b	5.6	7.1	6.23 ± 0.56a
2.	Tyrosol ^x^	13.4	0.40	1.21	3.0	4.8	4.00 ± 0.62a	3.2	5.9	4.60 ± 0.83a	5.1	6.6	5.94 ± 0.44b
3.	3,4-DHPEA-EDA ^y^	22.4	0.94	2.86	76.4	93.5	85.00 ± 7.19a	26.2	68.1	45.40 ± 15.00b	31.5	36.1	34.4 ± 1.75c
4.	Pinoresinol ^x^	25.2	0.16	0.47	nd	nd	nda	2.9	3.0	2.96 ± 0.05b	3.6	3.8	3.72 ± 0.08c
5.	Luteolin ^x^	25.7	0.46	1.40	6.0	7.4	6.95 ± 0.25a	7.4	7.6	7.49 ± 0.09b	4.0	4.4	4.22 ± 0.15c
6.	*p*-HPEA-EDA ^y^	26.3	0.94	2.86	134.8	158.5	146.30 ± 8.40a	66.1	98.1	82.6 ± 11.01b	83.1	91.5	88.10 ± 2.96b
7.	Apigenin ^x^	28.2	0.36	1.10	1.7	1.9	1.81 ± 0.09a	2.5	2.6	2.55 ± 0.05b	1.5	1.7	1.61 ± 0.09c
8.	3,4-HPEA-EA ^y^	28.5	0.94	2.86	82.1	96.4	89.50 ± 5.36a	130.3	136.4	133.40 ± 2.40b	67.7	82.8	75.10 ± 5.95c

RT = retention time; Min = minimal value; Max = maximal value; Mean = average value; LOD = limit of detection; LOQ = limit of quantification; SD = standard deviation; nd = not detected (<LOD); ^x^ Identification based on RT and UV-Vis spectra of pure compounds; ^y^ Tentative identification by UV-Vis spectra and comparison of retention times with the literature data, and compounds dosed using the oleuropein calibration curve; 3,4-DHPEA-EDA, dialdehydic form of decarboxymethyl elenolic acid linked to hydroxytyrosol (decarboxymethyl oleuropein aglycon); *p*-HPEA-EDA, dialdehydic form of decarboxymethyl elenolic acid linked to tyrosol (decarboxymethyl ligstroside aglycon); 3,4-DHPEA-EA, oleuropein aglycon; The mean values within each row labeled with different letters are significantly different (Tukey’s test, *p* < 0.05).

## 3. Experimental Section

### 3.1. The Samples and Preparing of the Hydrophilic Fractions

All the olive oils were produced from the fruits from the Dalmatia region (Zadar hinterland, Croatia) in 2014. The olive trees were cultivated under same agronomic and agrotechnical conditions. 12-year-old olive trees were planted in squares (7 × 5 m spacing) in the same orchard (*ca.* 2 ha). There was no irrigation. Five batches of the healthy olive fruits (replicates; each with 200 kg of the fruits) were handpicked from each olive variety at the same maturity index (MI = 4.3). MI was calculated as a subjective evaluation of the skin color and flesh as proposed by Uceda and Frias [42]. The fruits of each variety were separately processed in the extraction plant Molinova TG (Gruppo Pieralisi, Pieralisi S.p.A. Jesi, Italy) within 24 h of collection. Before the extraction of each batch, the plant was cleaned. The fruits were crushed with a hammer crusher and olive paste was malaxed for 35 min at 26 ± 1 °C in the mixer. The olive oil was separated by centrifugation through two phase decanter (without addition of warm water). All oils were filtered through 25 mm GD/X 0.45 μm cellulose acetate filters (Whatman, Milan, Italy) and thereafter stored in dark glass bottles at 4 °C until the analyses.

Hydrophilic fractions (HFs) of the oils were prepared by adding 5 mL of CH_3_OH–H_2_O (80:20 v/v) mixture in 3 g of the oil placed in 20 mL screw cap test-tube. The mixture was blended in an ultrasonic bath for 15 min at 30 °C (the emulsion was allowed to separate). The hydrophilic layer was placed in a round flask. The oil extraction was repeated two times; the hydrophilic extracts were combined and evaporated on a rotary vacuum evaporator at 30 °C. The residue was dissolved up to 5 mL with the 80:20 CH_3_OH–H_2_O solution and filtered through a Whatman 13 mm GD/X 0.2 μm cellulose acetate syringe filter (Whatman, Milan, Italy).

### 3.2. Determination of the Oils Basic Characteristics

Free acidity (% of oleic acid (%18:1)), peroxide value (mEq O_2_/kg of the oil) and UV absorption characteristics (K_232_ and K_270_) were determined according to the European Union Commission Regulations EC 1989/2003 [13] as in our previous paper [23]. K_232_ and K_270_ were determined with an UV spectrophotometer (Specord 200, Analytik Jena AG, Jena, Germany) at 232 and 270 nm using 1% solution of the oil in cyclohexane and a path length of 1 cm. All parameters were determined in triplicate for each sample.

### 3.3. Headspace Solid-Phase Microextraction (HS-SPME)

HS-SPME was performed on SPME fiber (divinylbenzene/carboxen/polydimethylsiloxane; DVB/CAR/PDMS) purchased from Supelco Co (Bellefonte, PA, USA). The fiber was conditioned according to Supelco Co instructions before the extraction. VOO (5 g) was placed in 15 mL glass vial and sealed with PTFE/silicone septa. The vial was placed in a water bath at 40 °C for equilibration (15 min) and extraction (40 min) under constant stirring with a magnetic stirrer (1000 rpm). After sampling, the fiber was inserted into the injector (250 °C) of GC-FID/MS for 6 min for thermal desorption of the volatiles into the GC column.

### 3.4. Gas Chromatography and Mass Spectrometry (GC-FID/MS)

An Agilent Technologies (Palo Alto, CA, USA) gas chromatograph (7890A) with flame ionization detector, mass selective detector (5975C) and HP-5MS capillary column (5%-phenyl)-methylpolysiloxane Agilent J & W GC column; 30 m, 0.25 mm i.d., coating 0.25 μm) was utilized. Helium was applied as carrier (1.5 mL/min). The injector operated in split mode (2:1 split ratio) at 260 °C. The column was heated at 40 °C for 3 min, thereafter to 100 °C (5 °C/min) and later to 260 °C (3 °C/min) and then held to 260 °C for 3 min. The MS conditions were: source temperature 230 °C; quadrupole at 150 °C; transfer line at 270 °C; EI 70 eV and *m*/*z* 29–350. The peaks were identified by comparison of the retention indices with authentic samples (relative to C_9_-C_25_ *n*-alkanes) and literature as well as by comparing their mass spectra with Wiley 9 MS library (Wiley, New York, NY, USA) and NIST08 (Gaithersburg, MD, USA) database. The percentage composition was computed from the peak areas using the normalization method (without correction factors). The component percentages (Table 2) were calculated as mean values from duplicate GC-FID analyses of each sample.

### 3.5. Liquid Chromatography with Diode Array Detector (HPLC-DAD)

HF phenolic compounds were detected and quantified with the HPLC-DAD method described by Tuberoso *et al.* [43]. A ProStar HPLC system (Varian Inc., Walnut Creek, CA, USA) was employed equipped with a pump module 230, an autosampler module 410, a ThermoSeparation diode array detector SpectroSystem UV 6000lp (Thermo Separation, San Jose, CA, USA) and a Gemini C18 column (150 × 4.60 mm, 3 μm, Phenomenex, Casalecchio di Reno, BO, Italy). The mobile phase was 0.2 M H_3_PO_4_ (solvent A) and CH_3_CN (solvent B) at a constant flow rate (1.0 mL/min), mixed (linear gradients) as follows: at 0 min A:B ratio 85:15 (v/v), reaching 60:40 (v/v) in 30 min, then 40:60 (v/v) in 10 min and finally at 100% B until 50 min. Prior to each injection the system was 10 min stabilized with A:B ratio 85:15 (v/v). The injection volume was 10 μL. The phenols analysis was carried out at 280 nm (hydroxytyrosol, tyrosol, vanillic acid, pinoresinol, oleuropein, and ligstroside derivatives) and 360 nm (luteolin and apigenin). Oleuropein and ligstroside derivatives were tentatively identified according to the literature data [44,45]. The obtained chromatograms and spectra were elaborated with a ChromQuest V. 2.51 data system (ThermoQuest, Rodano, Milan, Italy). Stock standard solutions were prepared in CH_3_OH and working solutions in CH_3_OH–H_2_O (80:20, v/v). The method was validated according to the International Conference on Harmonization of Technical Requirements for Registration of Pharmaceuticals for Human Use (ICH) guidance note [46] as previously reported [23]. All compounds were dosed using the calibration curve constructed with corresponding standard, except oleuropein and ligstroside derivatives that were dosed using the oleuropein calibration curve. The correlation values were comprised between 0.9992 and 0.9999.

### 3.6. Targeted Tocopherols Analysis

A Shimadzu LC System (Shimadzu, Milan, Italy) with a SCL-10A VP system control, a LC-10AD VP binary pump, a SIL-10AD VP autoinjector, connected to a Jasco 821-FP spectrofluorometer detector (Jasco Europe, Cremella, LC, Italy) was used. The detector operating conditions were λ_ex_ = 298 nm and λ_em_ = 325 nm. The injection volume was 20 μL. Separation was obtained with a Gemini C18 column (150 × 4.6 mm, 3 μm; Phenomenex) using CH_3_CN–CH_3_OH (90:10, v/v) at 0.8 mL/min. The oil (10 mg) was placed in 1.8 mL vial and 200 μL of CHCl_3_ was added as well as 790 μL of CH_3_CN–CH_3_OH mixture (50:50, v/v); the homogenization was performed with a vibration mixer. α- and γ-tocopherols standard solutions were prepared in CH_3_COCH_3_, while working solutions were prepared to appropriate dilution with the eluent mobile phase. Linearity in the range 0.1–6 mg/kg was 0.9998.

### 3.7. Determination of Total Chlorophylls and Carotenoids

The oil solutions of 5% (w/w) in CH_3_COCH_3_ were prepared and absorbances were measured (at 464 nm for carotenoids and at 669 nm for chlorophylls) on a 10 mm quartz cuvette utilizing a Varian Cary 50 UV-Visible spectrophotometer (Varian, Leini, TO, Italy). Chlorophyll a and β-carotene stock standard solutions were prepared in CH_3_COCH_3_, as well as working solutions that were prepared with proper dilutions (0.1–2.0 mg/kg, r = 0.9997 and 0.02–0.50 mg/kg, r = 0.9994 for chlorophyll a and β-carotene, respectively).

### 3.8. Folin-Ciocalteu Assay

HF total phenolic content was determined spectrophotometrically with a modified Folin-Ciocalteu method [47]. Shortly, HF (100 μL) was added to Folin-Ciocalteu phenol reagent (500 μL). After 5 min, 3 mL of 10% Na_2_CO_3_ (w/v) was added, the mixture was shaken, and thereafter diluted with H_2_O to a final volume of 10 mL. After a 90 min incubation period at room temperature, the absorbance was read at 725 nm (against a blank) on a 10 mm optical polystyrene cuvette (Kartell 01937, Kartell Spa Noviglio, Mi, Italy) utilizing a Varian Cary 50 spectrophotometer (Varian, Leini, TO, Italy). The total polyphenol content expressed as mg/kg of gallic acid equivalent (GAE) were obtained using a calibration curve of a freshly prepared gallic acid standard solution (5–100 mg/kg, r = 0.9999).

### 3.9. DPPH Assay

HF antiradical activity was assessed with the DPPH spectrophotometric method and the obtained data were expressed as Trolox equivalent antioxidant capacity (TEAC) [47]. HF (50 μL) was dissolved in 2 mL of 0.04 mmol/L DPPH in CH_3_OH. Spectrophotometric readings were carried out at 517 nm with a Varian Cary 50 spectrophotometer using 10 mm optical polystyrene cuvette after an incubation period of 60 min in dark at room temperature. A Trolox calibration curve (0.02–1.00 mM) was prepared (r = 0.9997) and data were expressed in TEAC (mmol/kg).

### 3.10. Statistical Analysis

Statistical analysis by SPSS 17.0 statistical software (SPSS Inc., Chicago, IL, USA) was applied to datasets to perform descriptive multivariate statistical study. The mean values were compared using the Tukey’s honestly significant difference test (*p* < 0.05).

## 4. Conclusions

In continuation of our research on monovarietal extra VOOs from Croatian autochthonous olive varieties, it was established that *Oblica*, *Lastovka* and *Levantika* VOOs exhibited all characteristics within the limits for the extra VOO category. Among the headspace volatiles, the most abundant was (*E*)-hex-2-enal. (*Z*)-hex-3-enal was not found in *Lastovka* oil and was different abundant in *Levantika* and *Oblica* oils and therefore useful for their differentiation. *Levantinka* oil did not contain hexanal. The results showed α-tocopherol dominance in all samples followed by γ-tocopherol. The lowest value of γ-tocopherol was measured in *Lastovka* oil. Higher total pigment content (clorophylls + carotenoids) was found in *Lastovka* oil. The most abundant secoiridoids were the dialdehydic forms of elenolic acid linked to hydroxytyrosol or tyrosol, particularly 3,4-DHPEA-EDA, *p*-HPEA-EDA and 3,4-HPEA-EA, which dominated *Oblica* oil, and 3,4-HPEA-EA in *Lastovka* oil. Different abundance of these compounds could be useful to distinguish olive oils from different cultivars.
